# Laryngeal cancer relative survival trends from 1972 to 2021 in the Nordic countries

**DOI:** 10.2340/1651-226X.2024.40823

**Published:** 2024-08-04

**Authors:** Rayan Nikkilä, Aaro Haapaniemi, Timo Carpén, Eero Pukkala, Antti Mäkitie

**Affiliations:** a Department of Otorhinolaryngology – Head and Neck Surgery, University of Helsinki and HUS Helsinki University Hospital, Helsinki, Finland; b Research Program in Systems Oncology, Faculty of Medicine, University of Helsinki, Helsinki, Finland; c Finnish Cancer Registry, Institute for Statistical and Epidemiological Cancer and Research, Helsinki, Finland; d Department of Oral and Maxillofacial Surgery, Lahti Central Hospital, Päijät-Häme Joint Authority for Health and Wellbeing, Lahti, Finland; e Palliative Care Unit, Comprehensive Cancer Center, University of Helsinki and HUS Helsinki University Hospital, Helsinki, Finland; f Health Sciences Unit, Faculty of Social Sciences, Tampere University, Tampere, Finland; g Division of Ear, Nose and Throat Diseases, Department of Clinical Sciences, Intervention and Technology, Karolinska Institute and Karolinska Hospital, Stockholm, Sweden

**Keywords:** Laryngeal cancer, larynx, relative survival, head and neck cancer

## Abstract

**Background and purpose:**

Changes in treatment approaches, characterised by the shift from laryngectomy to a focus on organ-preserving methods may have potentially resulted in lower survival. We aim to identify differences in survival trends for laryngeal cancer (LC) in the Nordic countries over a period of 50 years, and discuss the potential impact of factors such as changes in treatment protocols.

**Materials and methods:**

Five-year relative survival (RS) data from 1972 to 2021 were obtained from the NORDCAN database 2.0 which included 33,692 LC cases, of which 85% were diagnosed among men. In the NORDCAN database, the age-standardised RS is calculated using the Pohar Perme estimator with individual International Cancer Survival Standards weights. Joinpoint regression models were used to assess potential shifts in trend over the years in RS.

**Results:**

While Denmark and Norway demonstrated an increasing trend in 5-year RS from 1972 to 2021, in Finland and Sweden, the 5-year RS among men remained static, without any discernible significant trend. Over the 30-year period from 1992–1996 to 2017–2021, RS improved by 9, 4, 13, and 2 percentage points in Denmark, Finland, Norway, and Sweden, respectively. Among women in Sweden, a linear negative trend was observed, noticeable as a 16 percentage-point decline in 5-year RS from the earliest to the latest period.

**Interpretation:**

The underlying causes for the differences in survival trends remain unclear. Besides differences in treatment protocols, several other factors can affect RS making the interpretation of RS trends challenging.

## Introduction

According to estimates of the Global Cancer Observatory (GLOBOCAN) nearly 190,000 cases of laryngeal cancer (LC) – 88% among men – were diagnosed in 2022 [1]. Furthermore, the global number of new cases is expected to rise by approximately 60% by the year 2045. The incidence rates exhibit geographical variation: for example, while Finland, Norway, and Sweden display some of the lowest incidence rates in Europe (age-standardised rate [ASR] World 0.69 to 0.98/10^5^), Eastern and Southern Europe is affected by the highest incidence rates worldwide (ASR World 2.9 to 5.1/10^5^) [1]. Mostly diagnosed between the ages of 50 and 70 years, squamous cell carcinomas account for over 95% of all LCs [2, 3]. In the United States (US), in 2017, glottic cancers constituted approximately 75%, supraglottic cancers 23%, and subglottic cancers 2% of all LCs [4].

While originally, efforts to treat LC patients focussed on aggressive surgery – mainly total laryngectomy – nowadays, a multidisciplinary approach aims at organ preservation – and particularly the preservation of vocal function – by combining surgery, radio-, and chemotherapy [4, 5]. While total laryngectomy continues to be essential for treating the most advanced tumours (T4) and recurrent disease, transoral laser surgery, partial laryngeal resection and radiotherapy/chemoradiotherapy are used for less advanced tumours (T1–3) resulting in less late effect morbidity [6].

Survival for LC varies markedly depending on the age of the patient, tumour’s stage and anatomical location, and treatment protocol [4]. Changes in treatment approaches, characterised by the shift from laryngectomy to a focus on organ-preserving methods have been hypothesised to result in lower survival rates [7]. However, besides these, it is also important to consider several other factors that may affect relative survival (RS) estimates, when interpreting treatment outcome. Our aim is to describe secular trends in RS from 1972 to 2021 in four Nordic countries – Denmark, Finland, Norway, and Sweden – using data from the NORDCAN database [8].

## Materials and methods

We obtained all data from the NORDCAN database 2.0 of the Association of the Nordic Cancer Registries [8]. Five- year RS for LC (ICD-10 codes C10.1 and C32) in Denmark, Finland, Norway, and Sweden from 1972 to 2021 were extracted using the NORDCAN tools. We do not show results for Iceland, as cases were too few.

NORDCAN contains cancer statistics of the Nordic countries, namely Denmark, Finland, Iceland, Norway, and Sweden. Tabulated data on cancer incidence, mortality, prevalence, and RS are freely available, sourced from national cancer registries and cause of death registries. National cancer registration was initiated in 1943 in Denmark, in 1953 in Finland and Norway, and in 1958 in Sweden. Cancer registries receive information on cancer cases from general and specialist practitioners, hospitals, and pathology departments, with some exceptions [9]. All countries, except Sweden, also receive information from death certificates whenever cancer is mentioned. Utilising population registries and unique personal identity codes enables the tracking of each cancer patient until death or migration, facilitating the calculation of RS.

In the NORDCAN database, the age-standardised RS is calculated using the Pohar Perme estimator with individual International Cancer Survival Standards (ICSS) weights for age-standardisation [10–12]. The expected survival is derived from age, sex, and calendar-specific rates within each population. For the analysis of five-year calendar periods, age is categorised into five groups (– 0–49 years, 50–59 years, 60–69 years, 70–79 years, and 80–89 years). The corresponding weights assigned to these age groups are 0.12, 0.17, 0.27, 0.29, and 0.15, respectively. Patients with zero survival, patients aged 90 or higher, and cases based on information only from death certificates, were excluded from survival calculations. Only patients’ first primary LC was included.

We used joinpoint regression models to assess whether a positive or negative shift is observable in the 5-year RS, which may be reflective of treatment protocol changes. The dependent variable was the RS and the independent variable was the time period. The 5-year time periods were numerically encoded to represent time sequentially (1–10). Joinpoint regression analysis was not conducted for Finnish and Norwegian women because of missing RS data for some 5-year periods. Slope, standard error (SE), and *P*-value, which tests whether the slope is significantly different from zero, are reported. The slope in this context represents the rate of change in RS in percentage points per 5-year period. We performed all analyses using the R software (The R Project for Statistical Computing) version 4.2.2 using the *segmented* package. Survival plots were created with the utilisation of the *ggplot2* package.

## Results

The NORDCAN database included 33,692 cases of LC diagnosed from 1972 to 2021 in Denmark, Finland, Norway, and Sweden (Table 1). Of all cases, 85% (*n* = 29,021) were diagnosed among men. Data validity was consistent across the countries: of all cases, 99% were microscopically verified (98% in Denmark, 99% in Finland and Norway, and 100% in Sweden).

**Table 1:** Number of laryngeal cancer cases diagnosed in Denmark, Finland, Norway, and Sweden from 1972 to 2021 by country and period.

Period	Men					Women				
	Denmark	Finland	Norway	Sweden	All	Denmark	Finland	Norway	Sweden	All
1972–1976	782	644	370	933	**2,729**	155	44	35	97	**331**
1977–1981	940	618	466	949	**2,973**	155	65	57	106	**383**
1982–1986	1,024	627	538	950	**3,139**	216	57	58	112	**443**
1987–1991	1,029	533	525	881	**2,968**	259	61	57	134	**511**
1992–1996	1,065	517	521	823	**2,926**	217	53	100	119	**489**
1997–2001	987	526	534	758	**2,805**	220	74	99	156	**549**
2002–2006	1,023	502	523	810	**2,858**	230	56	79	149	**514**
2007–2011	1,101	536	482	760	**2,879**	231	72	93	166	**562**
2012–2016	1,015	529	493	737	**2,774**	235	70	102	146	**553**
2017–2021	985	573	454	714	**2,726**	209	103	92	176	**580**
**All periods**	**9,951**	**5,605**	**4,906**	**8,315**	**28,777**	**2,127**	**655**	**772**	**1,361**	**4,915**

*Source*: NORDCAN database 2.0.

### Men

**Figure 1:**
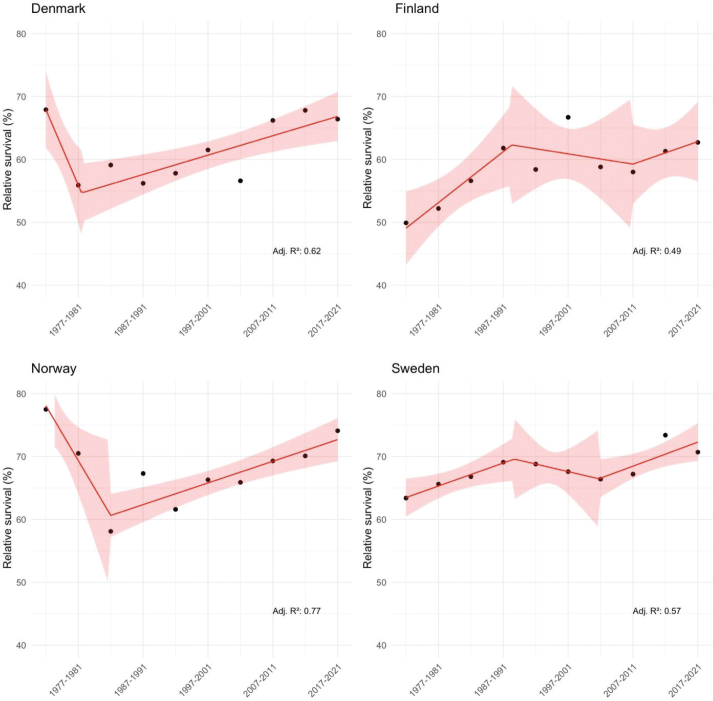
Joinplot analysis with 95%-confidence intervals and adjusted R-square values (Adj. R²) for 5-year age-standardised relative survival among **men** with laryngeal cancer, by 5-year periods of diagnosis.

From 1972–1976 to 1992–1996, RS decreased by 10 and 16 percentage points in Denmark and Norway, respectively, and increased by 9 and 5 percentage points in Finland and Sweden, respectively (Supplementary Table 1). Subsequently, from 1992–1996 to 2017–2021, RS improved by 9, 4, 13, and 2 percentage points in Denmark, Finland, Norway, Sweden, respectively. In the latest period, 2017–2021, 5-year RS was 66% (95% CI: 62%–71%), 63% (95% CI: 58%–68%), 74% (95% CI: 69%–80%), and 71% (95% CI: 67%–75%) for Denmark, Finland, Norway, and Sweden, respectively. Changing trends in RS for each country are illustrated by the joinplot analysis in Figure 1.

In Denmark, the RS at the earliest period in 1972–1976 was 68%. A noticeable decline to 56% in 1977–1981, followed by a gradual increase to 66% in the latest period (2017–2021) was evident. Indeed, the joinpoint regression analysis for Denmark revealed a significant shift in trends after 1987–1991: following an initial decrease in RS from 1972–1976 to 1977–1981 (slope –12.0, SE 4.38, *P*-value 0.034), the trend reversed to increase to a RS of over 65% in the last three periods (slope 1.54, SE 0.48, *P*-value 0.018). Excess in the last three periods was only seen in 80-89-year-old patients whose 5-year RS in those periods was approximately 80%, much higher than in the earlier periods (e.g., 42% in the period 2001-2006)

In Finland, no statistically significant changes in trends were observed. The RS increased gradually from 50% in 1972–1976 to 62% in 1987–1991 (slope 4.06, SE 1.58, n.s.) and remained between 58% and 63% until 2017–2021.

The RS of 78% in Norway in the first period (1972–1977) was the highest across all countries and periods. The RS in Norway then fell to 58% in 1982–1986, marking the lowest point (slope –8.72, SE 3.84, n.s.). Afterward, a positive increasing trend is observable (slope 1.72, SE 0.42, *P*-value 0.006) with RS constantly exceeding 65% in the last five periods from 1997 to 2021.

In Sweden, no statistically significant changes in trends were observed. Starting in the earliest period (1972–1976), the RS was 63%, followed by a gradual increase in subsequent periods (slope 1.83, SE 0.82, n.s.), reaching 69% in 1987–1991, and remaining between 69% and 73% until 2017–2021.

### Women

From 1972–1976 to 1992–1996, RS decreased by 1 and 9 percentage points in Denmark and Norway, respectively, and increased by 4 percentage points in Sweden (Supplementary Table 2). Subsequently, from 1992–1996 to 2017–2021, RS improved by 9 and 16 percentage points in Denmark and Norway, respectively, and decreased 20 percentage points in Sweden. In the latest 10-year period, 2012–2021, 5-year RS was 66% (95% CI: 63%–70%) for Denmark, 62% (95% CI: 58%–66%) for Finland, 70% (95% CI: 66%–75%) for Norway, and 72% (95% CI: 68%–76%) for Sweden.

**Figure 2:**
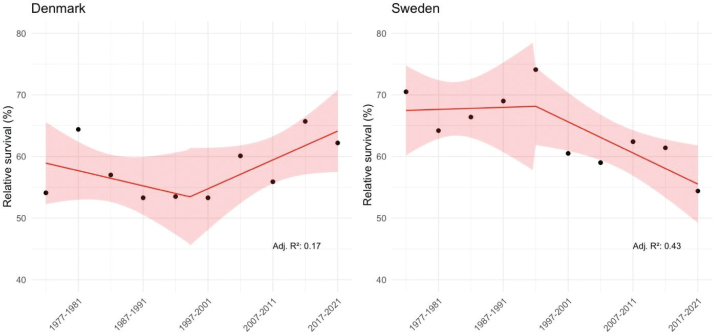
Joinplot analysis with 95% confidence intervals and adjusted R-square values (Adj. R²) for 5-year age-standardised relative survival among women with laryngeal cancer by 5-year period of diagnosis.

Joinpoint analysis for Denmark and Sweden is illustrated in Figure 2 (NORDCAN did not report 5-year RS for all 5-year periods in Finland and Norway because of insufficient number of cases). While the joinpoint analysis does not reveal any statistically significant changes in trends over time in RS among Danish women, Sweden showed a significant linear negative trend (slope –1.38, SE 0.49, *P*-value 0.022), supportive of the observable decline in 5-year RS from 71% in earliest period to 55% in the latest.

## Discussion

While Denmark and Norway demonstrated an increasing trend in 5-year RS of LC over the last decades, in Finland and Sweden, the 5-year RS among men remained static between 1987 and 2021, without any discernible significant trend. Additionally, a negative trend in 5-year RS was observable among women in Sweden, noticeable as a decline in 5-year RS over time from 71% in 1972–1976 to 55% in 2017–2021.

Zitricky et al. [13], utilising the same NORDCAN 2.0 database, previously described a less positive development in 5-year survival for LC than in other head and neck cancers. The static trends for LC patients observed in Finland and Sweden among men in the current study mirror previous findings observed in other countries. Overall, in Europe, RS for LC has remained stagnant from 1999–2001 to 2005–2007 [14]. In England, survival among LC patients also remained at a plateau from 1991 to 2006 for both genders, with only minor gains observed in 5-year survival, which were primarily attributed to increased survival of glottic LC patients [15]. In Japan, from 1993 to 2011, LC showed only a slight improvement in 5-year RS remaining between 74% and 79% throughout the years [16]. Similarly, in Ontario, Canada, overall and laryngectomy-free survival among LC patients have remained unchanged between 1995 and 2007 [17]. In the US, LC patients’ survival has not changed noticeably over the past few decades. In the US, Hofman et al. [7] noted that the estimated 5-year RS decreased from 66% in the period of 1975–1977 to 63% in 2005–2011. The authors argued that the decreasing survival trend may be attributable to a shift in treatment protocols favouring organ-preserving techniques. Furthermore, in the US, between 2004 and 2016, Li et al. [18] observed no notable improvement in 5-year RS which remained within the range of 62% to 64. Relative survival estimates published from different countries are not necessarily directly comparable because of, among other reasons, differences in population demographics, discrepancies in cancer registration and reporting methods, and lack of age-standardisation or, if age-standardisation has been done, differences in the weights assigned to the different age groups – not often reported by studies. In the studies of Hofman et al. [7] and Li et al. [18] conducted in the US, RS was calculated using the National Cancer Data Base, which collects data from approved hospitals. As it excludes some hospitals and their patients, the database is thus not representative of the entire patient population, especially if patients receiving non-curative treatment are excluded (for instance, about 2%–5% of patients with advanced LC are assigned to palliative treatment at diagnosis in the Nordic countries [19, 20]).

Over the last few decades, significant breakthroughs have impacted the diagnostics and treatment of LC and other head and neck cancers, which would assumably lead to enhanced RS over time. Improved imaging techniques – such as positron emission tomography (PET) aiding in the detection of metastatic cancers and narrow-band imaging enhancing endoscopic examinations [21, 22] – may lead to more precise classification of tumours by more accurately identifying the extent of tumour spread or neck metastases, and thus more aggressive treatment, conducive ultimately to better RS. Improved diagnostic techniques would also be expected to detect tumours at an earlier stage and thus also improve RS. In the US, Li et al. [18] reported no improvement in RS from 2004 to 2016, and suggested an increase in the proportion of stage IV tumours as a cause. Unfortunately, we do not have accurate comparable data on disease stage across time and countries. However, in Denmark, Norway, and Sweden, no major changes in the distribution of TNM stages for LC could be observed across the three five-year periods from 2004 to 2016 [23].

Treatment modalities for LC have also considerably evolved. Indeed, the latter half of the 20th century saw a gradual shift from open partial laryngeal surgeries – such as supraglottic laryngectomy or supracricoid laryngectomy – to transoral laser surgery, which is mainly indicated for early-stage glottic and supraglottic cancers (T1, T2), but is also used in the treatment of advanced LCs [24, 25]. In addition to recent advancements in surgery, clinical trials since the 1990s have validated the efficacy of radiotherapy in treating early glottic and supraglottic LC while preserving laryngeal function [26]. Furthermore, intensity-modulated radiation therapy (IMRT), introduced after 2000, has been associated with oncological outcomes that are as good as, or superior to, those of conventional radiotherapy, while causing fewer side effects [27]. Regarding advanced LC, published in 1991, a landmark randomised controlled trial (RCT) found no difference in overall survival between laryngectomy compared with induction chemotherapy plus radiotherapy [28]. In 2003, a randomised trial demonstrated that radiotherapy with concurrent administration of cisplatin provided superior locoregional control and laryngeal preservation than induction chemotherapy followed by radiotherapy, or radiotherapy alone [29]. Consequently, chemoradiotherapy emerged as an alternative to laryngectomy in treating advanced stages of LC, with different implementation periods in the Nordic countries.

Denmark and Norway have experienced positive RS trends since the 1970s, whereas in Finland and Sweden, RS has remained stagnant. Over the 30-year period from 1992–1996 to 2017–2021, RS improved among men by 9 and 13 percentage points in Denmark and Norway, respectively, but only by 4 and 2 percentage points in Finland and Sweden, respectively. This variation might stem from differences in treatment approaches across countries. For example, between 1983 and 2010, chemoradiotherapy usage in Norway was 1% for glottic and 9% for supraglottic patients, compared to Finland (2001–2005) where it was 11% and 29%, respectively [19, 20, 30]. Denmark initiated chemoradiotherapy with cisplatin for stages III and IV LC only in 2007–2008 [31]. In Denmark, surgery for glottic cancer patients was minimal, at 1% between 1971 and 2000, increasing slightly to 4% from 2000 to 2011 [32]. Finland, Norway, and Sweden reported higher surgery rates for glottic LC, with Finland at 41% (2001–2005), Norway at 37% (1983–2010), and Sweden at 22% (2000–2014). Supraglottic cancer surgery rates also varied, with 51% in Finland, followed by 17% in Norway, and 11% in Sweden in their respective periods. Total laryngectomies – as primary or salvage treatment – were performed in a total of 6% of patients in Finland during 2001–2005, compared to 17% in Norway (1983–2010) and 16% in Sweden (2000–2014) [19, 20, 30]. These data – while not directly comparable – may hint at distinctive treatment strategies across these countries, with Finland employing surgery and chemoradiotherapy more frequently. The higher utilisation chemoradiotherapy in Finland might have led to an increased occurrence of toxicity.

Laryngeal cancer patients bear a higher burden of comorbidities, particularly cardiovascular diseases, which are strongly associated with tobacco smoking [33, 34]. Over the last decades, the treatment of cardiovascular diseases has evolved significantly with advances in medication and surgical techniques. Innovations, such as statins and antiplatelet-therapy, have improved management of cardiovascular diseases, while procedures like angioplasty and bypass surgery have enhanced surgical outcomes. These advancements in treating comorbidities may have contributed to better survival among LC patients, especially for those with less-advanced tumours, [35–37] contributing over time to a positive RS trend.

Besides differences in treatment protocols, several other factor may affect RS estimates, which can further complicate their interpretation. Over the past few decades, the incidence of LC among men in the Nordic countries has been declining along with decreasing prevalence of smoking [38]. It can be hypothesised that the proportion of glottic tumours, which are strongly associated with tobacco smoking, has decreased and the proportion of supraglottic tumours, known for poorer survival outcomes – for instance, 5-year RS of 79% versus 47% for glottic and supraglottic cancers in Ontario, Canada [17] – has increased. Notably, Finland had the highest prevalence of heavy smokers in 1965, with the strongest reduction across the Nordic countries in this group occurring from 1965 to 1985 [39]. Consequently, this might have led to an increase in the proportion of supraglottic tumours especially in in Finland, potentially resulting in a negative or static survival trend. However, smoking rates have also been decreasing across other Nordic countries during the last decades [40]. Therefore, substantial differences in glottic/supraglottic tumour distribution, which would explain the differing RS trends, seem unlikely. Moreover, according to previous data, the proportions of glottic and supraglottic tumours during 1983–2010 in Norway and during 2001–2005 in Finland were similar [19, 20].

As life expectancy in the general population rises, so does the expected survival. This increase would decrease the RS if the observed survival of LC patients does not increase at the same rate. Therefore, a rise in general life expectancy could lead to a decline in RS for LC, if all other factors among the LC patient population remain constant. From 1990 to 2017, life expectancy increased by 6.6 years in Denmark, 7.6 years in Finland, 7.1 years in Norway, and 5.8 years in Sweden [41]. Given that these increases have been similar across the countries, it does not account for why the 5-year RS for men has increased in Norway and Denmark but remained static in Finland and Sweden over the past decades.

It is also crucial to consider methodological factors that may affect the RS across countries. For instance, the Swedish Cancer Registry’s data collection differs from other Nordic registries, as it does not use death certificates as a data source nor conduct follow-back searches in other registers for cancer cases identified from death certificates. This tends to result in lower coverage of cases with short survival times and thus higher survival estimates as compared to the other Nordic countries. It is important to note that age-standardised RS may not reflect the actual RS of a given patient population. Age-standardised RS can be higher or lower than the actual RS depending on the population’s age structure. RS among LC patients is lowest for those over 75; therefore, a higher proportion of older patients results in a lower RS [42]. In NORDCAN’s RS data, the weights for the 70–79 and 80–89 age group are 0.29 and 0.14, respectively, meaning 44% of patients are assumed to be 70 or older. From 2012 to 2021, the proportion of patients over 70 at diagnosis was 42% in Denmark, 43% in Finland, 50% in Norway, and 54% in Sweden. Hence, the RS without age-standardisation for Norway and Sweden would be lower. Although age-standardised RS may not be equal to true RS of cancer patients, age-standardisation removes the influence of different age distributions, enabling fairer comparisons between countries and across time periods.

While our study presents insights into cancer RS across Nordic countries, our study lacked data on stage and subsite of LC and treatment data, which hampers us to discern the effects of specific treatments on survival. Still, a key strength of our study lies in our utilisation of the NORDCAN database 2.0, which aggregates detailed cancer statistics from Denmark, Finland, Iceland, Norway, and Sweden, and with over 99% of registered LC cases included in the survival analyses microscopically verified. Cancer registries in the Nordic countries are known for their hight data completeness [43–46]. For instance, in Finland, completeness was 95% for head and neck cancers between 2009 and 2013, and it was estimated to be even higher in the 1980s [45]. In Norway, a study reported virtually perfect completeness for head and neck cancers from 1953 to 1991 [47]. Denmark transitioned from manual to automatic coding in 2008 [43], which coincided with a decrease in the incidence rates of various cancers. It is well possible that also LC cases have not been registered. Should a large proportion of these unregistered cases involve patients with short survival times, this could result in overestimated RS compared to earlier periods. It appears likely that the substantial rise in RS of the oldest LC patients between the periods before 2007 and the period 2002-2021 - which explains the whole increase in RS in Denmark – is related to the change in the cancer registration system. As the NORDCAN database covers the entire Nordic population, it offers more accurate and representative RS estimates than those based on hospital registers. Joinpoint regression analysis enabled us to assess whether any shift in trend is observable in RS over time. The joinpoint regression evaluates the collective pattern of data across all time points, pooling data from all periods, rather than comparing individual period estimates in isolation, with *P*-values focussing on the slope of the relationship between survival and time.

To conclude, the 5-year age-adjusted RS for male LC patients in Finland and Sweden has remained stagnant since the 1990s. Conversely, the 5-year age-adjusted RS among men in Denmark and Norway have seen improvements, aligning with levels observed during 1972–1981. While this study provides valuable insights into secular RS trends of LC patients at the population level in the Nordic countries, the reasons for the differences in RS trends observed and for the decreasing trend in 5-year RS among women in Sweden remain unanswered. Changes in treatment approaches, marked by the transition away from laryngectomy towards a focus on organ-preserving methods, may have resulted in improvement in patients’ quality of life [48], though not necessarily in survival. However, the actual impact of treatment changes on RS remains unclear, as multiple factors can affect RS, which makes the interpretation of RS trends challenging. To comprehensively assess the effects of treatment changes, a multicentre cohort study would be essential to analyse disease-specific survival of LC patients, accounting for treatment variables and confounding factors.

## Supplementary Material

Laryngeal cancer relative survival trends from 1972 to 2021 in the Nordic countries

## Data Availability

Data are available on https://nordcan.iarc.fr/en.
