# Supplementary material for: Laryngeal cancer relative survival trends from 1972 to 2021 in the Nordic countries
Source: Acta Oncol. 2024 Aug 4;63:40823. doi: 10.2340/1651-226X.2024.40823 (PMC11332480; doi:10.2340/1651-226X.2024.40823)
Supplement: Laryngeal cancer relative survival trends from 1972 to 2021 in the Nordic countries [file AO-63-40823-s1.pdf]

Supplementary material has been published as submitted. It has not been copyedited, or typeset by Acta Oncologica

Supplementary table 1. Five-year age-standardized relative survival (%) with 95% confidence interval for laryngeal cancer among **men** by 5-year period of diagnosis (Source: NORDCAN database 2.0).

	Denmark	Finland	Norway	Sweden
1972-1976	67.9[61.5-75.0]	49.9[43.0-57.9]	77.5[68.4-87.8]	63.4[58.7-68.3]
1977-1981	55.9[50.9-61.4]	52.2[46.9-58.1]	70.5[63.2-78.7]	65.6[61.2-70.4]
1982-1986	59.1[54.4-64.3]	56.6[50.7-63.3]	58.1[52.3-64.5]	66.8[62.2-71.8]
1987-1991	56.2[51.7-61.2]	61.8[55.2-69.2]	67.3[61.4-73.8]	69.1[64.6-73.9]
1992-1996	57.8[53.1-62.8]	58.4[52.4-65.1]	61.6[55.7-68.1]	68.8[64.1-73.9]
1997-2001	61.5[56.9-66.4]	66.7[59.5-74.8]	66.3[60.9-72.2]	67.6[63.1-72.4]
2002-2006	56.6[52.3-61.2]	58.8[52.8-65.5]	65.9[60.3-71.9]	66.4[62.0-71.0]
2007-2011	66.2[61.9-70.7]	58.0[52.6-63.8]	69.3[63.9-75.2]	67.2[62.8-72.0]
2012-2016	67.8[63.2-72.7]	61.3[55.7-67.5]	70.1[64.9-75.6]	73.4[69.0-78.0]
2017-2021	66.4[61.9-71.2]	62.7[57.6-68.2]	74.1[69.0-79.5]	70.7[66.5-75.1]

Supplementary table 2. Five-year age-standardized relative survival (%) with 95% confidence interval for laryngeal cancer among **women** by 5-year period of diagnosis (Source: NORDCAN database 2.0).

	Denmark	Finland	Norway	Sweden
1972-1976	54.1[44.0-66.6]		65.2[47.5-89.5]	70.5[56.2-88.5]
1977-1981	64.4[52.9-78.3]	51.1[39.3-66.6]		64.2[50.5-81.6]
1982-1986	57.0[48.7-66.7]	63.7[48.4-83.7]	51.0[38.9-67.0]	66.4[55.5-79.5]
1987-1991	53.3[45.9-62.0]	47.0[35.1-62.9]	64.2[51.3-80.3]	69.0[59.4-80.1]
1992-1996	53.5[45.1-63.4]		56.5[45.6-69.9]	74.1[64.6-85.0]
1997-2001	53.3[45.6-62.4]		64.9[54.9-76.7]	60.5[51.8-70.7]
2002-2006	60.1[52.0-69.5]		68.6[57.6-81.8]	59.0[51.3-67.9]
2007-2011	55.9[48.6-64.4]	69.0[55.7-85.4]	58.9[50.5-68.7]	62.4[54.8-71.2]
2012-2016	65.7[57.8-74.7]		60.7[51.2-72.0]	61.4[52.5-71.8]
2017-2021	62.2[53.6-72.1]		72.1[61.9-83.9]	54.4[46.2-64.0]
